# Pleural Mesothelioma Presenting as Periumbilical Metastasis: The First Clinical Documentation

**DOI:** 10.1155/2013/198729

**Published:** 2013-04-18

**Authors:** R. F. Falkenstern-Ge, M. Kimmich, S. Bode-Erdmann, G. Friedel, G. Ott, M. Kohlhäufl

**Affiliations:** ^1^Division of Pulmonology, Klinik Schillerhoehe, Center for Pulmonology and Thoracic Surgery, Teaching Hospital of the University of Tuebingen, Solitude Street 18, Gerlingen, 70839 Stuttgart, Germany; ^2^Division of Pathology, Robert Bosch Krankenhaus, Teaching Hospital of the University of Tuebingen, Auerbachstrasse 110, 70376 Stuttgart, Germany; ^3^Division of Thoracic Oncology, Klinik Schillerhoehe, Center for Pulmonology and Thoracic Surgery, Teaching Hospital of the University of Tuebingen, Solitude Street 18, Gerlingen, 70839 Stuttgart, Germany; ^4^Division of Clinical Pathology, Robert Bosch Krankenhaus, Teaching Hospital of the University of Tuebingen, Auerbachstrasse 110, 70376 Stuttgart, Germany

## Abstract

*Introduction*. Pleural mesothelioma with metastasis to the subcutaneous tissue of the abdominal wall at first diagnosis and without penetration into the peritoneum is an extremely rare clinical presentation. *Methods*. Patients with pleural mesothelioma have low survival rate. Usually, the disease at presentation is confined to its site of origin (most often the pleural cavity). A 55-year-old man was referred to our center due to increasing dyspnea and a painful periumbilical mass in the anterior abdominal wall. CT scan revealed both advanced mesothelioma of the pleura and a tumor mass confined to the subcutaneous fatty tissue without penetration through the peritoneum. *Results*. Video-assisted thoracoscopy confirmed the diagnosis of epithelioid pleural mesothelioma, which was also confirmed by a biopsy of the periumbilical mass. Systemic chemotherapy with cisplatin and pemetrexed was initiated. Under the ongoing systemic chemotherapy, the evaluation revealed partial remission of pleura mesothelioma and its subcutaneous manifestation of the abdominal wall. *Conclusion*. Mesothelioma of the pleura with a simultaneous metastasis to the subcutaneous fatty tissue of the abdominal wall at presentation without penetration of peritoneum is a rare clinical presentation of mesothelioma disease. The knowledge of its natural history is very limited. This is the first ever clinical documentation of a patient with pleura mesothelioma and simultaneous subcutaneous manifestation of abdominal wall.

## 1. Main Article

**Figure 1:**
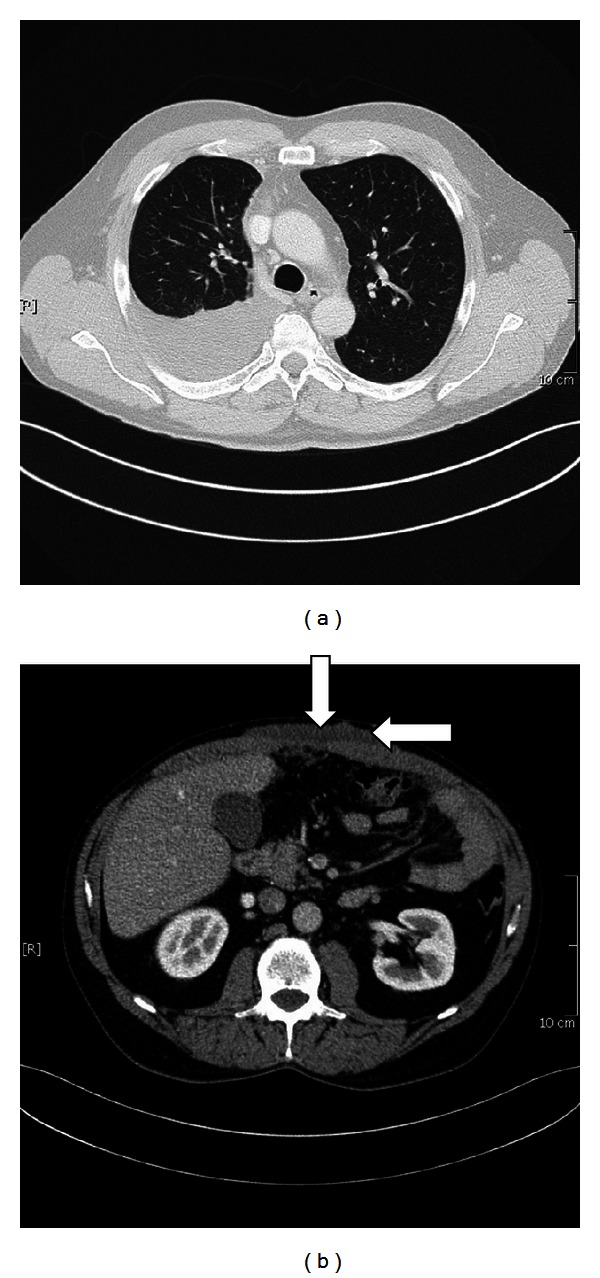
(a) CT scan shows a pleural effusion; the surface of the right lung is colonized and infiltrated by pleural mesothelioma. (b) Large tumor mass of the abdominal wall (10 × 7 cm) without penetration into the peritoneum.

A 55-year-old nonsmoker with a large pleural effusion at the right side was urgently referred to our center. He was occupied as a construction worker in the shipbuilding industry for a long time with asbestos exposure. He complained about increasing severe dyspnea and great abdominal pain. During initial physical examination we found a palpable periumbilical mass. The initial CT scan revealed pleural mesothelioma with right-sided effusion and a tumor mass in the abdominal subcutaneous fatty tissue (10 cm × 7 cm) without penetration through the peritoneum (Figures 1(a) and 1(b)). Video assisted thoracoscopy biopsies and biopsy of the periumbilical mass confirmed the histological diagnosis of epitheloid pleural mesothelioma (cT3N1 M1) with infiltration of the pleura and a simultaneous subcutaneous tissue metastasis of the abdominal wall without penetration into the peritoneum. 

**Figure 2:**
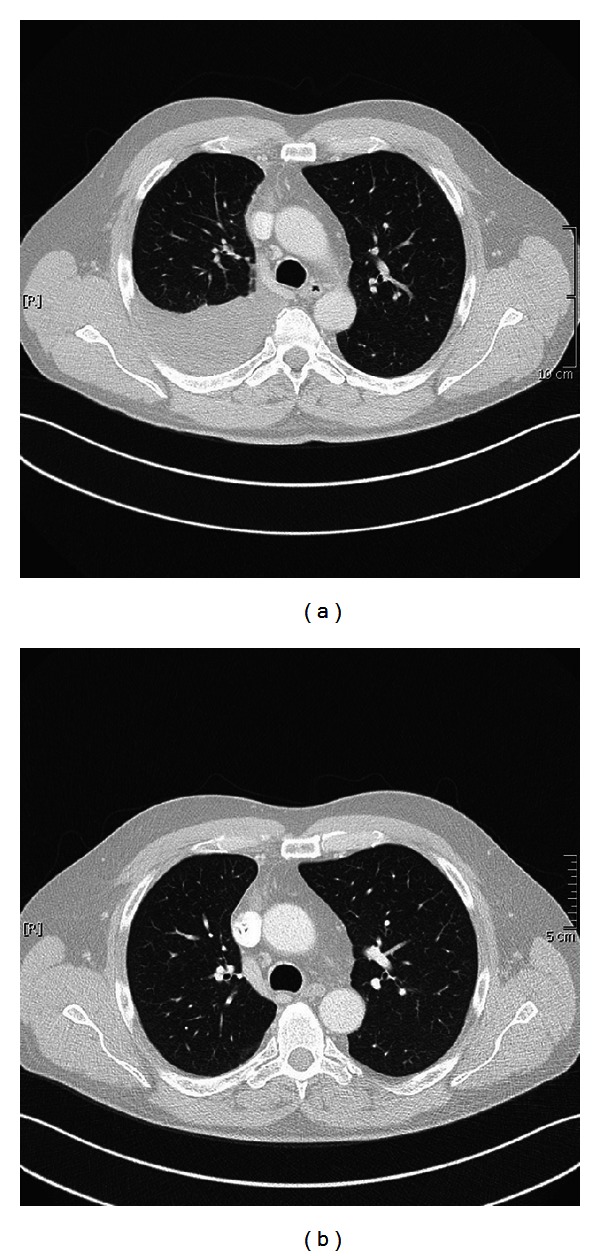
(a) Tumor manifestation before the systemic chemotherapy, (b) partial remission of the tumor in the right lung after 6 cycles of chemotherapy.

**Figure 3:**
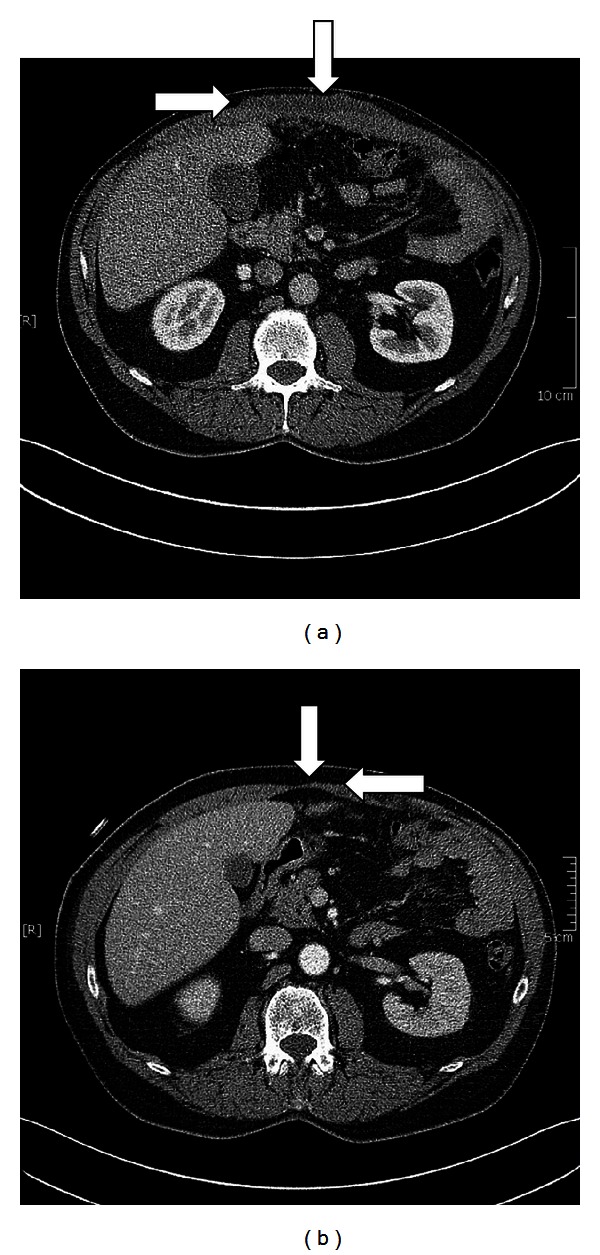
(a) Large subcutaneous metastasis of the abdominal wall before chemotherapy, (b) remission of the subcutaneous metastasis after 6 cycles of chemotherapy.

Due to the abdominal wall metastasis of the patient (cT3N1 M1), surgical option was not indicated. We initiated systemic chemotherapy with cisplatin and pemetrexed. The chemotherapy was well tolerated. Reevaluation with CT scan after 6 cycles of cisplatin and pemetrexed also showed partial remission of the pleural mesothelioma (Figures 2(a) and 2(b)) and remission of the manifestation in the abdominal wall (Figures 3(a) and 3(b)). The clinical conditioning of our patient improved clearly under chemotherapy; he was again partially employable.

## 2. Histopathology

**Figure 4:**
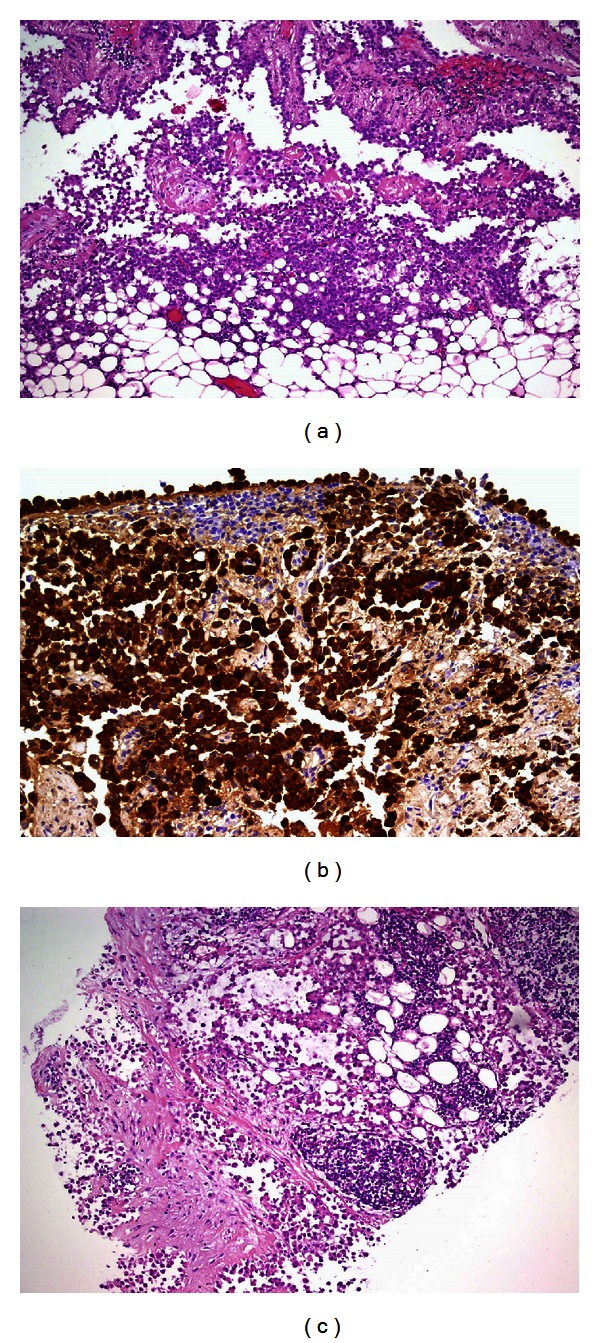
(a) The H&E staining ((a) ×200) of the pleural tumor. (b) The immunohistochemistry ((b) ×200) of the pleural tumor. (c) The abdominal tumor (H&E ×100).

Histopathological examination of biopsies gained on occasion of the video-assisted thoracoscopy showed an infiltration of the pleural fatty tissue by cohesive medium-sized cells with round even nuclei (Figure 4(a)). 

Immunohistochemistry proved the cells to express CK7, CK5/6, calretinin (Figure 4(b)), and WT1, whereas EA, TTF1, and Napsin were negative. Therefore, a diagnosis of malignant mesothelioma was rendered.

Examination of a punch biopsy from the abdominal mass revealed an identical tumor as seen in the pleural biopsies (Figure 4(c)). 

## 3. Discussion 

Malignant mesothelioma is a neoplasm originating from the mesothelial cells lining human body cavities. Mesotheliomas are aggressive tumors arising from various serous surfaces such as the pleura (65%–70%), peritoneum (30%), tunica vaginalis testis, or pericardium (1%-2%) [1, 2]. There is a long latency period between first exposure to asbestos and the diagnosis of mesothelioma that is rarely less than 15 years and often exceeds 30 years [3, 4]. The natural history of malignant pleural mesothelioma shows a median survival of 4 to 12 months of patients without intervention [5–7]. However, long-term survivors have been reported with a survival time of up to 19 years [2, 8]. 

As to the M staging, mesotheliomas have a lower rate of metastasis compared to non-small-cell-lung cancer (NSCLC), for example, into the cerebrum. Moreover, NSCLC metastasizes also more frequently into the skeleton, the kidneys, and the adrenal glands, whereas metastases of pleural mesotheliomas present more often as a diffuse peritoneal carcinosis [9].

In case of a pleural mesothelioma with peritoneal penetration, systemic chemotherapy with simultaneous intraperitoneal chemotherapy is considered to be the most effective therapeutic option [10]. Intraperitoneal chemotherapy can be instilled after surgery or without surgery through an abdominal catheter. At present, intraperitoneal application of cisplatin, doxorubicin, and paclitaxel combined with cytoreductive surgery is recommended [11].

Cisplatin is the most studied agent for pleural mesothelioma with or without peritoneal manifestations, which shows antitumor activity in 25% of patients [12]. Systemic chemotherapy with pemetrexed in combination with cisplatin has shown survival improvement in patients with pleural mesothelioma [12, 13]. 

Metastasis of mesothelioma of the pleura to the subcutaneous tissue is an extremely rare occurrence. Patel et al. reported on a 25-year-old woman, who had undergone chemotherapy, partial excision of tumor followed by radiotherapy of mesothelioma of the pleura, presented three months later with painless widespread subcutaneous nodules, histologically being diagnosed as metastases of mesothelioma of the pleura [14]. It is worth to be mentioned that those subcutaneous metastases were not simultaneous to the primary pleural mesothelioma during the first diagnosis.

To our knowledge of the current literature, this is the first reported clinical documentation of a pleural mesothelioma with a simultaneous solitary subcutaneous tissue metastasis of the abdominal wall without penetration of peritoneum at the time of diagnosis.
